# Circulating miRNA-21 and miRNA-155 Expression in Adult Patients With Bronchial Asthma and Its Association With Lung Function

**DOI:** 10.7759/cureus.111630

**Published:** 2026-06-27

**Authors:** Ram Niwas Jalandra, Kamla Kant Shukla, Srividhya Nandagopal, Rajani Kumawat, Saumya Shishir, Rasleen Kaur, Nishant Kumar Chauhan, Naveen Dutt

**Affiliations:** 1 Pulmonary Medicine, All India Institute of Medical Sciences, Bathinda, Bathinda, IND; 2 Biochemistry, All India Institute of Medical Sciences, Jodhpur, Jodhpur, IND; 3 Biochemistry, Jodhpur Institute of Engineering and Technology (JIET) Medical College and Hospital, Jodhpur, IND; 4 Biochemistry, All India Institute of Medical Sciences, Bathinda, Bathinda, IND; 5 Pulmonary Medicine, All India Institute of Medical Sciences, Jodhpur, Jodhpur, IND

**Keywords:** biomarkers, circulating, eosinophilic, gene expression, immune, inflammation, micrornas, molecular, serum ige, spirometry

## Abstract

Background & objectives: MicroRNAs (miRNAs) regulate post-transcriptional gene expression and have emerged as important contributors to the pathogenesis of asthma, as well as potential biomarker candidates. In this study, we evaluated the serum miRNA-21 and miRNA-155 expression in bronchial asthma and its association with lung function and inflammatory markers.

Methods: In this pilot case-control study, a total of 72 participants were enrolled, including 36 adults with spirometry-confirmed asthma and 36 healthy controls recruited from hospital staff and attendants without respiratory symptoms or history of allergic disease. Spirometry was also performed in controls to exclude occult airflow limitation. A predefined exploratory hypothesis was that circulating miRNA-21 and miRNA-155 expression would differ between asthma and controls and may correlate with inflammatory biomarkers and lung function. Serum miRNA-21 and miRNA-155 expression was quantified by quantitative real-time polymerase chain reaction. Between-group comparisons were performed using the Mann-Whitney U test, and correlations with serum IgE, absolute eosinophil count (AEC), forced expiratory volume in one second (FEV1) and forced vital capacity (FVC) were examined using Spearman’s rank correlation coefficient.

Results: miRNA-21 expression was significantly higher in patients compared to controls with median values of 0.191 (IQR 0.114-0.312) vs 0.054 (IQR 0.027-0.195), P=0.006. In contrast, miRNA-155 expression did not differ significantly between the groups (median 0.159 (IQR 0.068-0.363) vs 0.220 (IQR 0.107-0.338), P=0.240). Among correlation analyses, only miRNA-155 showed a significant positive correlation with serum IgE (Spearman’s ρ=0.654, P<0.001) and no significant association of miRNA-21 with the IgE level. Neither miRNA correlated significantly with AEC, FEV1 or FVC.

Interpretation & conclusions: Serum miRNA-21 was significantly upregulated in bronchial asthma, supporting its possible association with asthma-related immune dysregulation. Although miRNA-155 was not differentially expressed between groups, its positive association with serum IgE suggests a possible relationship with atopic inflammatory pathways. These findings suggest that circulating miRNAs may reflect asthma-related inflammatory activity; however, their clinical biomarker utility requires further validation.

## Introduction

Bronchial asthma is a heterogeneous chronic airway disorder characterised by variable airflow obstruction, persistent airway inflammation and structural alteration of the airway wall. Recent advances in biomarker-driven classification have identified distinct asthma endotypes, particularly type 2-high disease, in which eosinophils and IgE are commonly used clinical indicators. However, there remains a need for additional non-invasive molecular markers to enhance disease phenotyping and monitoring [1].

Asthma is a major global public health concern affecting individuals across all age groups. According to the Global Burden of Disease (GBD) 2021 estimates, approximately 260 million people worldwide are affected by asthma, accounting for nearly 4,36,190 deaths annually [2,3].

In India, asthma prevalence is also substantial, with estimates suggesting that about 35 million individuals are affected. Community-based studies report prevalence rates ranging from 2 to 6% in adults, with higher rates in urban populations [4]. The rising burden of asthma, particularly in low- and middle-income countries, underscores the need for improved understanding of disease mechanisms and identification of novel biomarkers for better diagnosis and management.

MicroRNAs (miRNAs) are small, non-coding RNA molecules that regulate gene expression at the post-transcriptional level. They have the capacity to modulate entire inflammatory pathways by simultaneously influencing multiple messenger RNAs [5]. In asthma, altered miRNA expression profiles have been associated with key pathological processes, including epithelial cell responses, T-helper cell differentiation, cytokine signalling, airway smooth-muscle function and airway remodeling [6,7]. Furthermore, emerging evidence highlights the significance of extracellular vesicle-derived and exosomal miRNAs as promising, minimally invasive biomarkers in respiratory disease, including asthma [8-10].

Among the various miRNAs implicated in asthma, miRNA-21 and miRNA-155 have been extensively studied due to their involvement in immune regulation and allergic inflammation [6,7]. Experimental studies have demonstrated that miRNA-21 is upregulated in allergic airway inflammation and can promote Th2-dominant responses via IL-12-related pathways [11]. Clinical studies have also reported elevated circulating miRNA-21 in individuals with asthma, suggesting its potential role in diagnosis and assessment of treatment response [12,13].

In contrast, the role of miRNA-155 appears to be more variable and context-dependent. Experimental data indicate that miRNA-155 plays a critical role in Th2-mediated eosinophilic airway inflammation and influences mast-cell activation through PI3Kγ-dependent pathways [14,15].

Several clinical studies have explored the association between circulating miRNAs with asthma-related parameters, including pulmonary function, inflammatory markers and therapeutic outcome [16,17]. A recent study demonstrated that miR-21-5p regulates airway inflammation and epithelial-mesenchymal transition by activating the PI3K/AKT signaling pathway, thereby contributing to airway remodeling and disease progression [18]. Additionally, inhibition of miRNA-21 in experimental models has been shown to attenuate airway inflammation and hyperresponsiveness, underscoring its pathogenic role in asthma [19].

Despite growing interest in circulating miRNAs in asthma, evidence from Indian adult patients remains limited, particularly regarding the relationship of serum miRNA-21 and miRNA-155 with spirometric parameters and inflammatory markers. Therefore, this pilot case-control study was designed to evaluate serum miRNA-21 and miRNA-155 expression in treatment-naïve adult patients with spirometry-confirmed bronchial asthma and to explore their association with serum IgE, absolute eosinophil count (AEC), forced expiratory volume in one second (FEV₁), and forced vital capacity (FVC).

## Materials and methods

This exploratory pilot study was conducted to generate preliminary data from an Indian adult cohort because limited regional data are available on circulating miRNA expression in asthma.

A formal sample size calculation was not performed because this study was designed as an exploratory pilot investigation intended to generate preliminary data from an Indian adult population. The sample size was determined based on feasibility, availability of treatment-naïve asthma patients, and laboratory resource considerations.

This was a hospital-based case-control pilot study conducted with the objective of evaluating the expression of circulating miRNA-21 and miRNA-155 in 36 treatment-naïve patients of bronchial asthma and 36 healthy controls, and furthermore, determining their association with lung function parameters, absolute eosinophil counts, and serum IgE levels.

Study setting

The study was conducted in the Department of Pulmonary Medicine at AIIMS Jodhpur, India between June 2019 and June 2021 after obtaining approval from the Institutional Ethics Committee (Ref No. AIIMS/IEC/2019-20/870). Written informed consent was obtained from all participants.

Participants

A total of 72 participants were enrolled: 36 asthma patients and 36 healthy controls.

Inclusion criteria (cases)

Patients aged ≥18 years and treatment-naïve bronchial asthma cases diagnosed on clinical history and spirometry demonstrating reversible airflow obstruction were included.

Diagnostic criteria

Spirometry was performed using a standard spirometer. Patients demonstrating obstructive ventilatory defect underwent bronchodilator reversibility testing. Asthma was confirmed if FEV₁ increased by ≥12% and ≥200 mL after bronchodilator administration, according to Global Initiative for Asthma (GINA) 2019 guidelines [20].

Healthy controls were recruited from hospital staff members and patient attendants. All controls underwent spirometry and subjects with abnormal spirometry, respiratory symptoms, smoking history, allergic disorders, or chronic systemic illness were excluded.

Controls

Serum samples were processed within 2 h of collection and stored at −80°C until analysis. Approximately 200 µL serum was used for RNA extraction. Patients with visibly haemolysed samples were excluded from analysis to minimise contamination by cellular miRNA.

Age- and sex-matched healthy individuals without respiratory disease or allergy were recruited as controls.

The miRNeasy Serum/Plasma Kit (Qiagen, Cat. No. 217184) and miScript II RT Kit (Qiagen, Cat. No. 218161) were used according to manufacturer instructions. RNA quality assessment was performed using NanoDrop spectrophotometry. Because of the low RNA concentration typically obtained from serum samples, formal RNA integrity assessment could not be reliably performed.

Sample collection

Approximately 5 mL of peripheral venous blood was collected from each participant in sterile plain vacutainer tubes. Blood samples were allowed to clot at room temperature and subsequently centrifuged at 3000 rpm for 10 minutes to separate serum. The separated serum samples were aliquoted and stored at −80°C until further molecular analysis.

RNA extraction and complementary DNA synthesis

Total RNA, including small RNAs, was extracted from serum samples using the miRNeasy Serum/Plasma Kit (Qiagen, Germany) according to the manufacturer’s protocol. The quantity and purity of the isolated RNA were assessed using a NanoDrop 2000 spectrophotometer (Thermo Fisher Scientific, USA) by measuring the absorbance ratio at 260/280 nm.

Reverse transcription of miRNAs into complementary DNA (cDNA) was performed using the miScript II RT Kit (Qiagen, Germany). This kit enables polyadenylation of mature miRNAs followed by reverse transcription using oligo-dT primers. The reaction mixture was prepared according to the manufacturer’s instructions and incubated in a thermal cycler to synthesise cDNA from the isolated RNA.

Expression of miRNA-21 and miRNA-155 by quantitative real-time PCR (qPCR)

The qRT-PCR was performed using Power Master Mix SYBR Green (Thermo Fisher, Waltham, MA) following the protocol: two minutes at 94°C at the start, followed by 38 cycles of 20s each at 94°C, then 30 s at 60°C, and one minute at 72°C, followed by a dissociation stage. The inverse log of the delta/delta CT was used to calculate the relative expression. PCR reactions were performed in duplicate according to laboratory protocol, and mean Ct values were used for analysis. Data were normalised to snRNA U6, which was used as an endogenous control gene. The 2^-ΔΔCt method was used to calculate relative miRNA expression. The amplification efficiency of miRNA-21, miRNA-155, and U6 assays was within the acceptable range (90-110%), supporting the validity of relative quantification using the 2^-ΔΔCt method.

Statistical analysis

Statistical analysis was performed using IBM SPSS Statistics for Windows, Version 29 (Released 2022; IBM Corp., Armonk, New York, United States). As this was an exploratory pilot study, a formal sample size calculation was not performed. Normality of continuous variables was assessed using the Shapiro-Wilk test together with visual inspection of histograms and Q-Q plots. Normally distributed variables (age, BMI, haemoglobin, FEV₁, FVC and FEV₁/FVC ratio), absolute eosinophil count and miRNA expression levels were analysed using the independent t-test and expressed as mean ± SD. Skewed variables such as serum IgE were analysed using the Mann-Whitney U test and expressed as median (IQR). Correlations were evaluated using Spearman's rank correlation coefficient. A two-sided P-value <0.05 was considered statistically significant.

## Results

Demographic data of asthmatic patients and healthy controls

The present study was conducted with a total of 72 participants, out of which 36 were asthmatic patients and the remaining 36 were healthy controls. The patient group included 26 (72%) male patients and 10 (28%) female patients, while the control group included 24 (66.6 %) male patients and 12 (33.3 %) female patients. Asthma patients demonstrated significantly higher serum IgE and absolute eosinophil counts along with significantly lower lung function parameters (FEV1, FVC% and FEV1/FVC ratio) while BMI and haemoglobin were comparable (Table 1).

**Table 1:** Baseline demographic, clinical, and laboratory characteristics of bronchial asthma patients and healthy controls. Data are presented as mean ± standard deviation (SD) or number (%), as appropriate. BMI: body mass index; FEV₁: forced expiratory volume in one second; FVC: forced vital capacity. *Median (IQR) used for serum IgE (immunoglobulin E) levels.

Parameters	Cases (n=36)	Control (n=36)
Age (y)	29.6±10.2	26.8±6.6
Gender: Male/Female	26 (72%)/10 (28%)	24 (66.6%)/12 (33.3%)
BMI (kg/m^2^)	22.8±4.6	21±3.1
Serum IgE (IU/mL)*	643.0 (643.0-1113.0)	64.5 (22.5-99.5)
Absolute eosinophil count (x10^9^cells/L)	0.39±27.5	0.19±12.2
Haemoglobin (%)	13.3±1.9	13.2±0.7
FEV1% predicted	78±15.4	85.4±12.2
FVC% predicted	79.0±12.8	89±10.0
FEV1/FVC ratio	72.0±10.8	80±10.1

Serum miRNA expression

**Figure 1:**
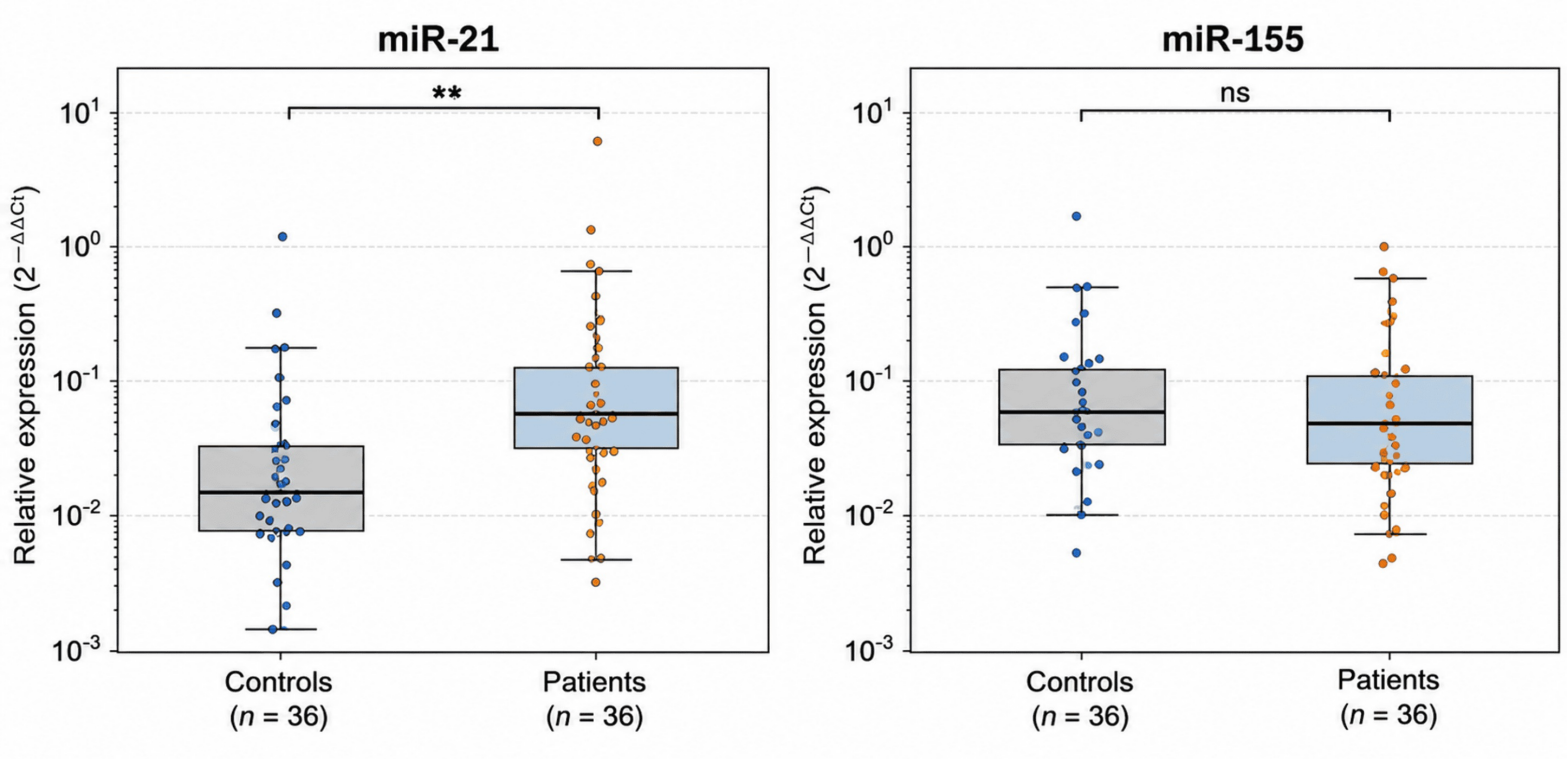
Relative expression of circulating miR-21 and miR-155 in patients with bronchial asthma and healthy controls. (A) Relative expression of miR-21 in controls and patients. (B) Relative expression of miR-155 in controls and patients. Boxplots represent the median (central line), interquartile range (box), and whiskers (1.5 × IQR). The y-axis is shown on a logarithmic scale for improved visualisation of skewed data. Statistical comparisons were performed using the Mann-Whitney U test. **P < 0.01; ns = not significant.

miRNA-21 expression was significantly higher in patients than in controls (median 0.191 (IQR 0.114-0.312) vs 0.054 (IQR 0.027-0.195); Mann-Whitney U = 253.5, P = 0.006). In contrast, miRNA-155 expression was not significantly changed between patients and controls (median 0.159 (IQR 0.068-0.363) vs 0.220 (IQR 0.107-0.338), P=0.240). Although the median patient value was lower than the control value, the distributions overlapped substantially, indicating the absence of a statistically robust between-group separation (Figure 1).

Correlation of miRNA expression with lung function and inflammatory markers

**Figure 2:**
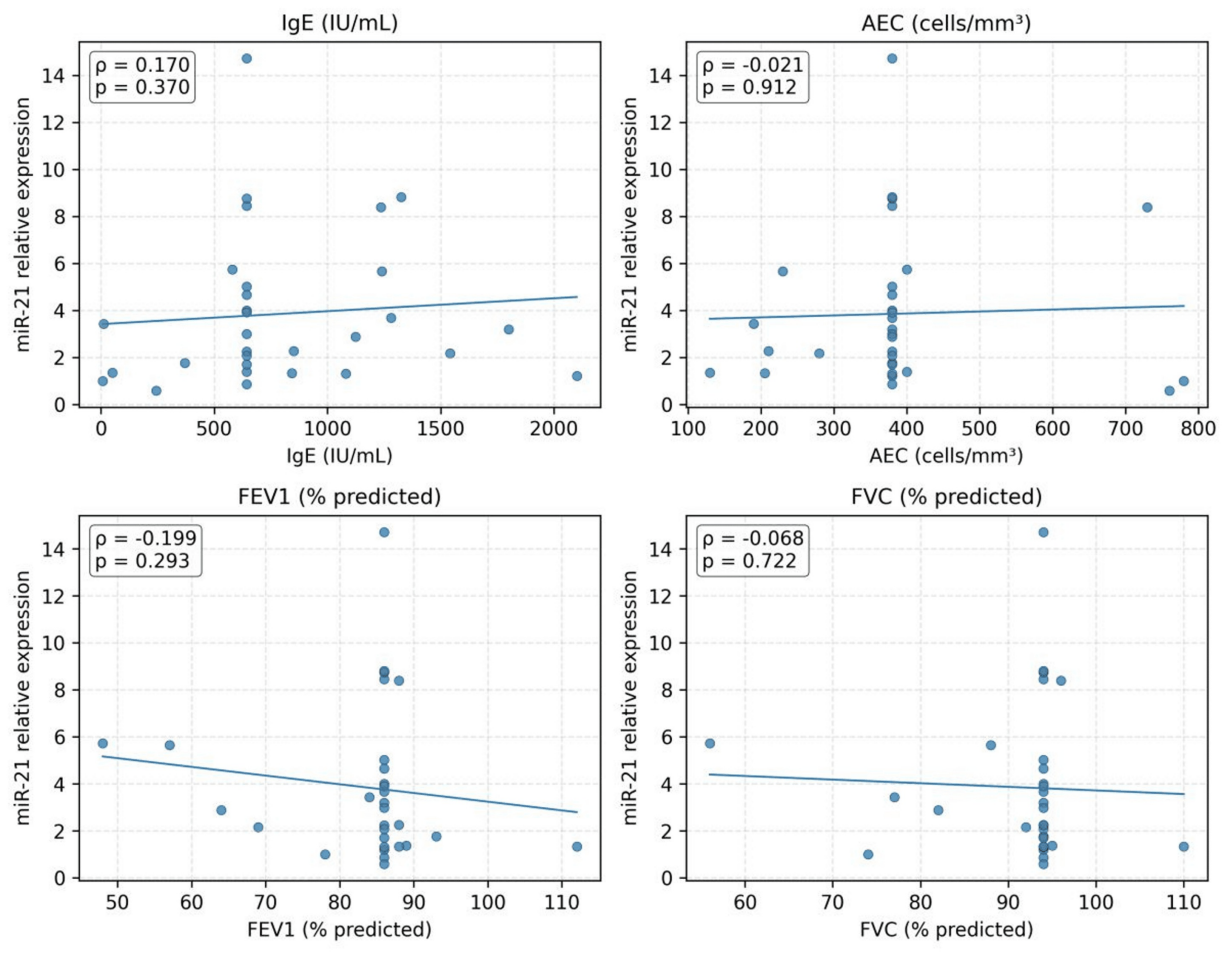
Correlation of circulating miR-21 expression with clinical parameters in patients with bronchial asthma. (A) Serum IgE. (B) AEC. (C) FEV₁ (% predicted). (D) FVC (% predicted). Spearman's correlation coefficient (ρ) and corresponding P-values are shown in each panel. miR-155: microRNA 155; IgE: immunoglobulin E; AEC: absolute eosinophil count; FEV₁: forced expiratory volume in one second; FVC: forced vital capacity.

**Figure 3:**
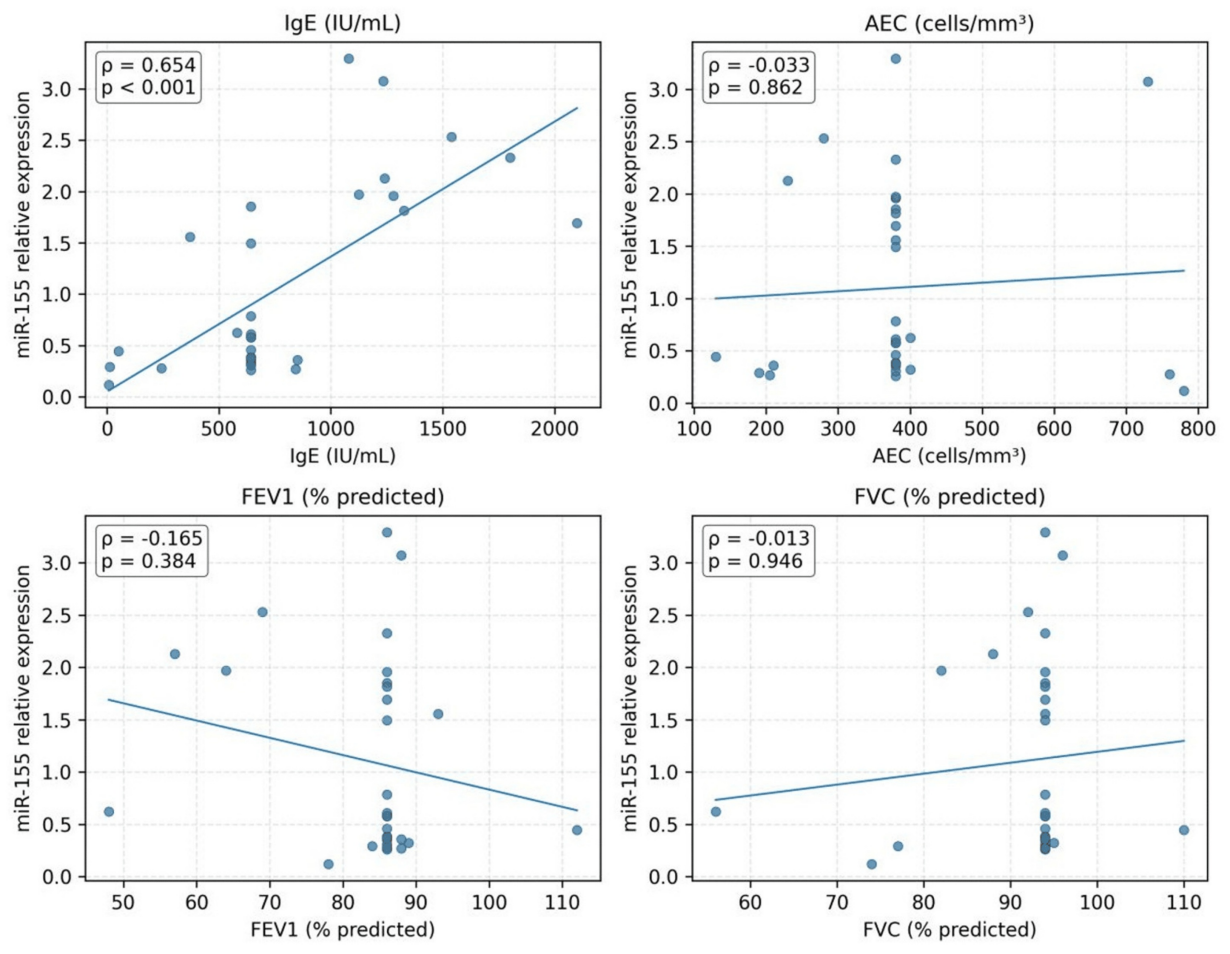
Correlation of circulating miR-155 expression with clinical parameters in patients with bronchial asthma. (A) Serum IgE. (B) AEC. (C) FEV₁ (% predicted). (D) FVC (% predicted). Spearman's correlation coefficient (ρ) and corresponding P-values are shown in each panel. miR-21: microRNA 21; IgE: immunoglobulin E; AEC: absolute eosinophil count; FEV₁: forced expiratory volume in one second; FVC: forced vital capacity.

miRNA expressions were correlated with the lung functions (FEV1 and FVC) and inflammatory markers (serum IgE level and AEC) in which only miRNA-155 showed a significant association with a clinical variable. Patient miRNA-155 relative expression correlated positively with serum IgE (Spearman’s ρ=0.654, P<0.001) (Table 2; Figure 2), suggesting linkage with an atopy/type 2 inflammatory milieu. No significant correlations were observed between miRNA-155 and AEC, FEV1 or FVC. Similarly, miRNA-21 relative expression did not show significant correlation with IgE, AEC, FEV1 or FVC (Table 2; Figure 3).

**Table 2:** Correlation of serum miRNA-21 and miRNA-155 expression levels with inflammatory and lung function parameters in patients with bronchial asthma. Correlations between serum miRNA expression levels and clinical variables were assessed using Spearman's rank correlation analysis. miRNA: microRNA; IgE: immunoglobulin E; AEC: absolute eosinophil count; FEV₁: forced expiratory volume in one second; FVC: forced vital capacity. *Indicates a statistically significant correlation (P < 0.05). The correlation between miRNA-155 expression and serum IgE levels was statistically significant (Spearman's ρ = 0.654, P < 0.001).

miRNA	Variable	n	Spearman’s ρ	P-value
miRNA-21	IgE (IU/mL)	36	0.170	0.370
	AEC (x10^9^cells/L)	36	-0.021	0.912
	FEV1 % predicted	36	-0.199	0.293
	FVC % predicted	36	-0.068	0.722
miRNA-155	IgE (IU/mL)	36	0.654	<0.001*
	AEC (x10^9^cells/L)	36	-0.033	0.862
	FEV1 % predicted	36	-0.165	0.384
	FVC % predicted	36	-0.013	0.946

## Discussion

The present study demonstrates that serum miRNA-21 expression was significantly increased in adults with bronchial asthma as compared to healthy controls, whereas miRNA-155 did not show a statistically significant difference. In addition, miRNA-155 expression showed a significant positive correlation with serum IgE, while neither miRNA displayed a significant relationship with spirometric indices (FEV1 & FVC) or AEC. These observations support the concept that circulating miRNAs may reflect systemic inflammatory activity more readily than lung function [14].

The higher miRNA-21 expression observed in our patients is biologically plausible and consistent with prior experimental and clinical work [16,17]. A 2009 study showed that miRNA-21 is upregulated in allergic airway inflammation and regulates IL-12p35, thereby favouring a Th2-predominant immune response [11]. Subsequent human studies reported elevated circulating miRNA-21 in asthma, including paediatric and adult cohorts, and proposed a role in diagnosis and response assessment [9,10]. Recent reviews also identified that miRNA-21 is one of the most reproducibly dysregulated asthma-associated miRNAs, which are linked with the inflammation, airway remodelling, and epithelial dysfunction [4,5].

Mechanistically, miRNA-21 has been shown to regulate the balance between Th1 and Th2 immune responses by suppressing IL-12/STAT4 signalling, which normally promotes Th1 differentiation. Inhibition of this pathway leads to enhanced Th2 polarisation and increased production of Th2 cytokines such as IL-4, IL-5, and IL-13, which are central mediators of allergic airway inflammation [21,22]. These cytokines stimulate IgE production, eosinophil recruitment, and mucus hypersecretion, thereby contributing to airway hyperresponsiveness and chronic inflammation. More recently, a study reported that circulating non-coding RNAs, including miR-21, are significantly elevated in patients with eosinophilic asthma and may serve as potential non-invasive biomarkers reflecting systemic inflammatory activity [23]. Furthermore, few studies have emphasised that miR-21 plays a central role in regulating multiple immune pathways involved in allergic airway inflammation and remodelling [22]. The significant increase in miRNA-21 expression observed in our study is therefore consistent with these findings and further supports its potential role as a molecular biomarker.

Interestingly, despite the significant upregulation of miRNA-21 in asthma patients, we did not observe a direct association between miRNA levels and spirometric parameters. This finding suggests that circulating miRNA levels may reflect underlying inflammatory pathways rather than directly correlating with functional measures of airway obstruction. Similar observations have been reported in previous studies, where circulating miRNAs were associated with inflammatory biomarkers but showed weak or inconsistent correlations with lung function indices [19]. These observations support the hypothesis that circulating miRNAs may be better indicators of underlying immune regulation or disease phenotype rather than lung function severity.

In contrast, with miRNA-155, we did not find any significant difference between cases and controls, despite its recognised mechanistic importance in allergic airway disease. This apparent discrepancy is not unexpected. Animal and cellular studies have shown that miRNA-155 can promote eosinophilic airway inflammation and mast-cell activation [11,12]. Many human studies have yielded more heterogeneous results, likely owing to differences in asthma phenotype, sample type, disease activity and treatment exposure [3,4,10]. A recent experimental study demonstrated that miRNA-155 modulates IL-13 signalling and airway epithelial immune responses in allergic airway inflammation [24]. Nevertheless, several clinical studies have reported only modest or non-significant differences in circulating miRNA-155 levels between asthma patients and healthy controls, particularly in stable disease states [13,25]. These discrepancies likely reflect differences in disease severity, inflammatory phenotype, treatment status, and biological sample source. The lack of a statistically significant increase in miRNA-155 expression in the present study may therefore be explained by several factors. First, circulating miRNA levels may vary depending on the inflammatory phenotype of asthma, particularly between eosinophilic and non-eosinophilic asthma. Second, differences in biological samples (serum vs airway tissue or sputum) may influence detected miRNA expression patterns. Finally, the relatively small sample size in the present study may have limited the statistical power to detect modest differences.

A notable finding of our study was the positive correlation between miRNA-155 and serum IgE. This is compatible with the broader literature linking miRNA-155 to type-2 immune responses, IgE-associated pathways and allergic inflammation [5,11,12]. By contrast, we did not observe significant correlations of miRNA-21 or miRNA-155 with FEV1 or FVC. Previous studies on circulating miRNAs and lung function have also shown variable results. A study found associations between certain circulating miRNAs and lung function in asthma, whereas another study found that correlations with clinical parameters may differ between allergic and non-allergic asthma [6,16]. A recent study had identified circulating miRNAs associated with bronchodilator response, further underscoring that individual miRNAs may relate to specific asthma traits rather than to baseline spirometric impairment alone [17].

Our findings suggest that miRNA-21 may be the stronger discriminatory biomarker for asthma-control separation in this cohort, whereas miRNA-155 may better reflect the atopic inflammatory milieu. This interpretation is also supported by reviews emphasising that circulating, extracellular-vesicle and exosomal miRNAs may capture distinct biological dimensions of asthma, including inflammation, remodelling, and treatment responsiveness [6,7].

Clinical implications

The findings of the present study highlight the potential role of circulating miRNAs as molecular biomarkers in asthma. The significant upregulation of miRNA-21 supports its potential utility as a non-invasive biomarker for asthma detection and monitoring of inflammatory activity. However, the lack of association between miRNA levels and lung function parameters suggests that miRNAs may reflect inflammatory pathways rather than direct functional impairment.

Strengths and limitations

This study integrates molecular biomarkers with clinical and inflammatory parameters, providing a comprehensive evaluation of asthma. The use of spirometry-confirmed cases and age- and sex-matched controls enhances internal validity. Standardised qRT-PCR methodology with appropriate normalisation and established analytical approaches ensures technical robustness. The study also contributes data from an Indian adult cohort, addressing a relative gap in regional evidence on circulating microRNAs in asthma.

However, certain limitations should be acknowledged. The relatively small pilot sample size limits statistical power for detecting modest associations. The cross-sectional design precludes causal inference. Circulating miRNA levels may not fully reflect local airway biology, as airway-derived samples were not analysed. Phenotypic stratification based on disease severity, atopy, or treatment status was not performed. In addition, key type-2 inflammatory cytokines were not evaluated, which could have strengthened the mechanistic interpretation.

Nevertheless, the present data add evidence from an Indian adult cohort and support further validation of serum miRNA-21 and miRNA-155 in larger phenotype-stratified studies. Future studies involving larger cohorts and airway-derived samples such as sputum or bronchoalveolar lavage fluid may provide deeper insights into the role of miRNAs in asthma pathogenesis. Another limitation of this study is that plasma miRNA levels were not evaluated, as miRNA expression analysis was performed exclusively using serum samples. Previous studies have shown that serum and plasma miRNA profiles may differ because of variations in sample processing and coagulation-related release of miRNAs. Future studies comparing both specimen types may provide additional insight into optimal biomarker selection.

## Conclusions

Serum miRNA-21 expression was significantly elevated in patients with bronchial asthma, suggesting a role in asthma-related inflammatory pathways. Although miRNA-155 expression did not differ significantly between groups, its positive correlation with serum IgE indicates a possible association with atopic inflammation. Neither miRNA was associated with spirometric parameters or eosinophil counts, suggesting that circulating miRNAs may reflect systemic inflammatory activity rather than airway function. These findings highlight the potential of circulating miRNAs as non-invasive biomarkers in asthma; however, the observed associations do not establish causality or diagnostic utility. As diagnostic performance was not evaluated, larger prospective studies with independent validation cohorts are needed to confirm these findings and clarify their clinical relevance.
